# Interplay Between the Unfolded Protein Response and Immune Function in the Development of Neurodegenerative Diseases

**DOI:** 10.3389/fimmu.2018.02541

**Published:** 2018-11-02

**Authors:** Paulina García-González, Felipe Cabral-Miranda, Claudio Hetz, Fabiola Osorio

**Affiliations:** ^1^Laboratory of Immunology and Cellular Stress, Program of Immunology, Institute of Biomedical Sciences, Faculty of Medicine, University of Chile, Santiago, Chile; ^2^Faculty of Medicine, Biomedical Neuroscience Institute, University of Chile, Santiago, Chile; ^3^Brain Health and Metabolism, FONDAP Center for Geroscience, Santiago, Chile; ^4^Program of Cellular and Molecular Biology, Institute of Biomedical Sciences, University of Chile, Santiago, Chile; ^5^Instituto de Ciências Biomédicas, Universidade Federal do Rio de Janeiro, Rio de Janeiro, Brazil; ^6^Buck Institute for Research on Aging, Novato, CA, United States; ^7^Department of Immunology and Infectious Diseases, Harvard School of Public Health, Boston, MA, United States

**Keywords:** UPR, neurodegeneration, immune system, inflammation, protein protein misfolding diseases, ER stress, immune cells, misfolded proteins

## Abstract

Emerging evidence suggests that the immune and nervous systems are in close interaction in health and disease conditions. Protein aggregation and proteostasis dysfunction at the level of the endoplasmic reticulum (ER) are central contributors to neurodegenerative diseases. The unfolded protein response (UPR) is the main transduction pathway that maintains protein homeostasis under conditions of protein misfolding and aggregation. Brain inflammation often coexists with the degenerative process in different brain diseases. Interestingly, besides its well-described role in neuronal fitness, the UPR has also emerged as a key regulator of ontogeny and function of several immune cell types. Nevertheless, the contribution of the UPR to brain inflammation initiated by immune cells remains largely unexplored. In this review, we provide a perspective on the potential role of ER stress signaling in brain-associated immune cells and the possible implications to neuroinflammation and development of neurodegenerative diseases.

## Introduction

### The unfolded protein response (UPR)

Proteostasis encompasses the dynamic interrelation of processes governing generation and localization of functional proteins (1). Physiological and pathological factors can impair the balance between protein load and protein processing, resulting into accumulation of improperly folded proteins (2, 3). Abnormal protein aggregation is a key feature of several neurodegenerative diseases, including Alzheimer's disease (AD), Parkinson's disease (PD), amyotrophic lateral sclerosis (ALS), Huntington's disease (HD) and prion-related disorders amongst others, collectively classified as protein misfolding diseases (PMDs) (4, 5).

**Figure 1:**
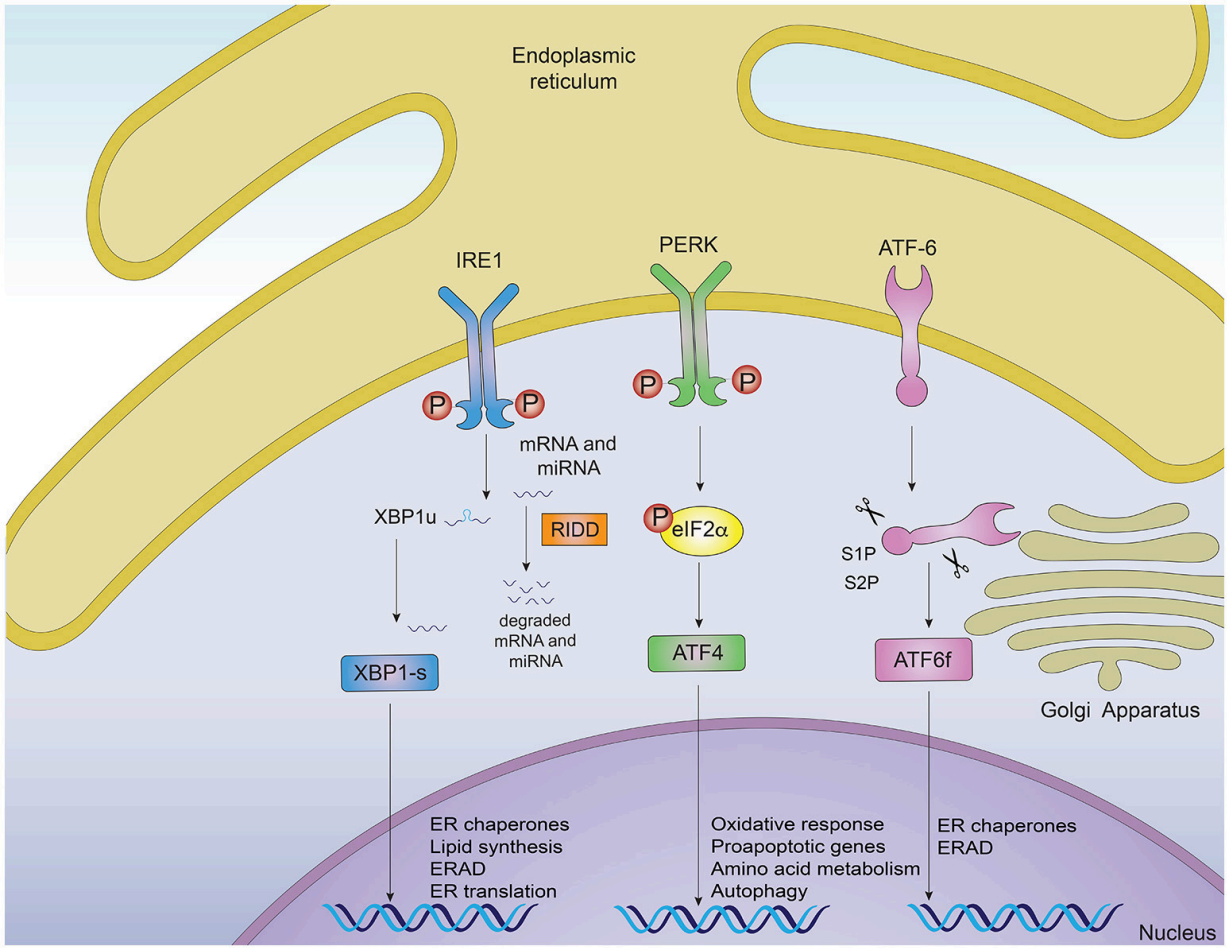
Signaling pathways of the unfolded protein response. Noxious stimuli in cells may induce endoplasmic reticulum (ER) stress and trigger an adaptive response known as the unfolded protein response (UPR), which is controlled by three main ER-resident sensors: IRE1α, PERK and ATF6. Upon ER stress, IRE1α autophosphorylates, leading to the activation of its RNase domain and the processing of the mRNA encoding for XBP1s, a transcriptional factor that upregulates genes involved in protein folding and quality control, in addition to regulating ER/Golgi biogenesis and ER-mediated degradation (ERAD). Additionally, IRE1α RNase also degrades a subset of specific RNAs and microRNAs, a process termed Regulated IRE1α-Dependent Decay (RIDD). The second ER sensor, PERK, phosphorylates the translation of the eukaryotic initiation factor eIF2α, decreasing the synthesis of proteins and the overload of misfolded proteins at the ER. PERK phosphorylation also leads to the specific translation of ATF4, a transcription factor that promotes the expression of genes related to amino acid metabolism, anti-oxidant response, autophagy and apoptosis. The third UPR sensor, ATF6, is a type II ER transmembrane protein that encodes a bZIP transcriptional factor in its cytosolic domain. Following ER stress, ATF6 translocates to the Golgi apparatus where it is processed, releasing a transcription factor which directs the expression of genes encoding ER chaperones, ERAD components and molecules involved in lipid biogenesis.

Protein misfolding is sensed by dedicated stress-response pathways that include the cytoplasmic heat shock response (HSR) and the unfolded protein response originated in the mitochondria and in the endoplasmic reticulum (ER) (3). Activation of these intracellular mechanisms by the presence of misfolded proteins leads to ameliorating the protein folding load and resolving proteotoxic stress (1, 3). In this context, the ER is a central node of the proteostasis network controlling folding, processing and trafficking of up to a third of the protein load in the cell (6). The UPR originated in the ER (for now referred as “UPR”) is a main intracellular mechanism responsible to safeguard the fidelity of the cellular proteome and for this reason, it will be the main focus of the current review (6, 7). The UPR is an adaptive reaction controlled by three ER-located signal transducers: inositol requiring enzyme 1 (IRE1) α and β, protein kinase R-like ER kinase (PERK) and activating transcription factor 6 (ATF6) alpha and beta (6) (Figure 1). Upon activation, these signal transducers activate gene expression programs through specific downstream transcription factors, restoring proteostasis and increasing ER and Golgi biogenesis (6, 8). IRE1α cleaves the mRNA encoding for the X-box binding protein (XBP1), removing a 26 nucleotide intron, which followed by RTCB (RNA 2′,3′-Cyclic Phosphate and 5′-OH ligase) ligation changes the coding reading frame, prompting the translation of a protein with transcription factor activity termed XBP1s (XBP1 spliced) (7). XBP1s controls the expression of genes involved in ER-associated degradation (ERAD), lipid biosynthesis, folding and quality control (9, 10). IRE1α RNase also directly degrades diverse mRNAs and microRNAs through a process termed “Regulated IRE1-Dependent Decay” (RIDD) (11), originally proposed to contribute to alleviating the detrimental effects of ER stress by reducing the protein folding load (12), in addition to regulating inflammation and apoptosis (13). Activation of PERK mediates protein translation shutdown via phosphorylation of eukaryotic initiation factor 2α (P-eIF2α), which also favors selective translation of certain mRNAs encoding proteins involved in cell survival, ER homeostasis and anti-oxidant responses, such as ATF4 and nuclear erythroid related factor 2 (NRF2) (6, 14). ATF6, translocates to the Golgi apparatus where it is cleaved by site-1 and site-2 proteases, releasing a transcription factor that directs the expression of genes encoding ERAD components, ER chaperones and molecules involved in lipid biogenesis (15, 16). XBP1s and ATF6 can also heterodimerize to control selective gene expression patterns (9). Moreover, the activity (signaling amplitude and kinetics) of the three UPR stress sensors is controlled by several cofactors through the assembling of distinct platforms termed the UPRosome (17). Binding of adapter proteins to the IRE1α UPRosome also mediates the crosstalk with other stress pathways including MAP kinases and NF-κB (6). Thus, the UPR integrates information regarding intensity and duration of the stress stimuli toward cell fate control in cells suffering from ER stress.

### UPR in brain homeostasis and protein misfolding diseases

ER stress signaling has a physiological as well as pathological role in brain function and development (18–20). In neurodegeneration, the UPR influences several aspects including cell survival, synaptic plasticity, axonal regeneration, protein aggregation and control of the secretory pathway (21–23). By mediating synthesis and secretion of the brain-derived neurotrophic factor (BDNF), XBP1s regulates neuronal plasticity at a structural, molecular and behavioral level (18, 24–27). Moreover, postmortem tissue analyses revealed that ER stress markers often co-localize with cells containing protein aggregates in brain of patients affected with PMDs (4, 5, 22, 28). In AD, the expression of Grp78/BiP, PDI and HRD1 is increased in the hippocampus and temporal cortex; and the phosphorylated forms of PERK, IRE1α and eIF2α are found in AD neurons and substantia nigra of PD patients (22, 29, 30). Phosphorylated IRE1α levels directly correlate with the degree of histopathological changes, where most cells showing neurofibrillary tangles exhibit signs of ER stress (31). Furthermore, ER stress signs are also observed in different brain areas in PD patients, a phenomenon also observed in incidental cases of subjects who died without PD symptoms but presented α-synuclein inclusions in the brain (32). Moreover, components of all UPR signaling branches are overexpressed in spinal cord samples of patients with familial and sporadic forms of ALS (33), as well as in striatum, parietal cortex and caudate putamen of HD and Prion disease patients (22, 34–39).

In support of a dual role of UPR in controlling cell fate in neurodegenerative diseases, genetic disruption and pharmacological intervention modulating ER stress signaling revealed that depending on disease type and the UPR component targeted, distinct and even opposite effects are observed [reviewed in (21, 40)]. Conditional deletion of XBP1 in the central nervous system (CNS) provides protective effects through upregulation of autophagy levels, improving motor performance in ALS, PD and Huntington's disease models (35, 37, 41), whereas XBP1 deficiency does not affect Prion pathogenesis *in vivo* (42). Ablation of IRE1α signaling in neurons decreases astrogliosis and amyloid β accumulation in an animal model of AD, correlating with improved neuronal function (31). Conversely, therapeutic gene delivery of active UPR components or ER chaperones to specific brain areas has shown outstanding effects in different animal models of PMDs (43). Different studies have shown that ectopic delivery of XBP1s into the hippocampus restored synaptic plasticity in an AD model (27), promoted axonal regeneration (44), reduced mutant huntingtin aggregation (45) and protected dopaminergic neurons against PD-inducing neurotoxins (41, 46).

Targeting the PERK pathway also provides contradicting results. PERK signaling supports oligodendrocyte survival in animal models of multiple sclerosis (MS) (47) and enhancement of eIF2α phosphorylation is protective in ALS and other models (32, 48), whilst ATF4 deficiency has a detrimental effect in spinal cord injury models, diminishing locomotor recovery following lesion, also impacting oligodendrocyte survival (49). Conditional deletion of PERK in the brain however, improved cognition in an AD model, correlating with decreased amyloidogenesis and restoration of normal expression of plasticity-related proteins (50, 51). Similarly, genetic targeting of CHOP has neuroprotective effects in a PD model, and ATF4 ablation protects against ALS (52, 53). Consistent with this, sustained PERK signaling has been shown to enhance neurodegeneration due to acute repression of synaptic proteins, resulting in abnormal neuronal function, as demonstrated through PERK inhibitors in Prion disease (54), frontotemporal dementia (48) and PD models (32). ATF6, on the other hand, protected dopaminergic neurons in another PD model, by upregulating ER chaperones and ERAD components (55, 56). Overall, UPR mediators have a pivotal role in the progression of various PMDs, nurturing the hypothesis that UPR components could be used as therapeutic targets in neurodegeneration (21, 22, 43).

### UPR in neuroinflammation

Immune surveillance is an active process in the brain. The mammalian CNS harbors several subtypes of leukocytes, which display physiological roles related to tissue homeostasis and regulation of the inflammatory response (57, 58). However, if unrestrained, inflammation can have detrimental effects in the CNS, contributing to the type of tissue malfunction that precedes pathological processes (59). During neuroinflammation, the immune response in the CNS is drastically altered, and it is typified by activation of resident microglia and invasion of peripheral immune cells into the parenchyma, including granulocytes, monocytes and, in pathologies like multiple sclerosis, lymphocytes (60–63). Interestingly, the UPR has shown to regulate inflammation in peripheral tissues, emerging as an interesting candidate for targeting CNS-associated inflammation in a field that remains largely unexplored. Thus, in addition to the well-described role of the UPR in neuronal fitness, it is also plausible that UPR activation in CNS-associated immune cells could contribute to modulating PMD development.

**Figure 2:**
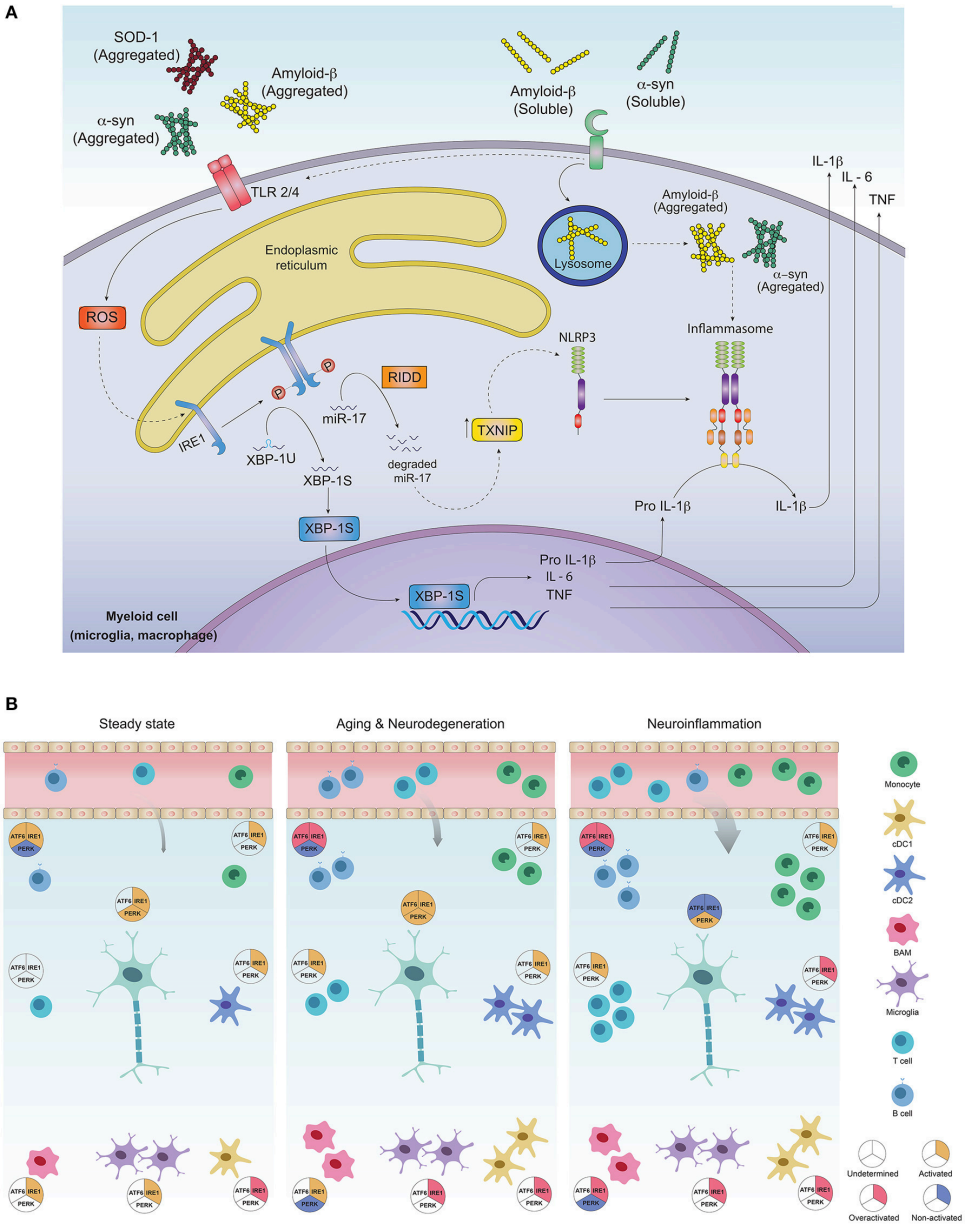
Activation of the unfolded protein response in CNS-residing immune cells may contribute to neuroinflammation and PMDs development. **(A)** Protein aggregates can promote inflammation via triggering of innate receptors and activation of the UPR. Neurodegeneration associated molecular patterns (NAMPS) such as protein aggregates are recognized by pattern recognition receptors (TLRs and PRRs) present on immune cells and signal through ROS production, which in turn could activate the IRE1α/XBP1s axis for co-transcriptional activation of IL-6, TNF and IL-1β. On the other hand, through RIDD, IRE1α induces degradation of the TXNIP-destabilizing microRNA mir-17, allowing activation of the NLRP3 inflammasome and processing of IL-1β into its active form. **(B)** Most of the immune cell lineages residing in the healthy and pathogenic brain are known targets of the UPR in peripheral tissues. In **steady state**, the most abundant immune cells in the brain are microglia, which along with border associated macrophages (“BAMs”) and dendritic cells act as sentinels, sampling the environment and clearing cell debris, maintaining CNS homeostasis. Except for dendritic cells and macrophages, which exhibit IRE1α/XBP1s activation, little is known about UPR activation in additional myeloid subsets, although microglia, macrophages and monocytes could potentially activate this axis downstream of PRR signaling. While very rare, B and T cells have been identified in the steady state brain, and activation of IRE1α/XBP1s has been proposed to be critical for their differentiation and activation. ATF6 axis is also necessary for B cell development and activation whilst absence of PERK contributes to plasma cell differentiation and immunoglobulin synthesis. Basal activation of UPR in neurons is still a matter of debate in literature as the function of IRE1α and PERK pathways has just begun to be understood in the context of normal neuronal physiology (72). In **aging and neurodegeneration**, the number of immune cells within the brain increases, due to higher cell activation as well as blood brain barrier infiltrates. Extracellular protein aggregation promotes activation of immune cells via PRRs, in addition to inducing ER stress and activation of the UPR, mainly the IRE1α/XBP1s axis. Microglia and dendritic cells become more activated, with higher production of pro-inflammatory and oxidative mediators and loss of their protein clearance function. This is further aggravated by antibodies against CNS-derived antigens by B cells accumulated in the CSF, mediated by the activation of IRE1α and ATF6 signaling. Activation of infiltrating T cells reactive to α-synuclein, amyloid-β and myelin constituents further amplify inflammation, resulting in more protein aggregation and neuronal loss. In neurons, UPR triggering may elicit both, adaptive or neurodegenerative responses, since all three UPR pathways are engaged in brain diseases and have been found to be altered during the normal aging process. Different inducers of **neuroinflammation**, have shown to engage the UPR in neurons and promote a greater inflammatory response due to immune cell infiltration, mainly B and T cells. The cDC1 subset of dendritic cells could activate IRE1α for cross presentation of antigens to infiltrating CD8^+^ T cells, and cDC2 as well as monocyte-derived DCs may set an inflammatory environment through cytokine secretion and activation of infiltrating CD4^+^ T cells. Macrophages and microglia also become highly activated and could tune IRE1α/XBP1s upon recognition of NAMPs. Inflammatory mediators such as cytokines prime axonal destruction and neuronal loss. It remains to be addressed weather UPR triggering in these cells corresponds to a homeostatic (adaptive) response, or a terminal (neurodegenerative) response due to sustained unresolved ER stress.

One hallmark of neuroinflammation is the presence of tumor necrosis factor (TNF), interleukin (IL)-1β, and IL-6 in brain, cerebrospinal fluid (CSF) and serum of patients with AD, PD and HD (63–65). Production of pro-inflammatory cytokines across tissues depends on activation of innate immune sensors (known as pattern recognition receptors, PRRs) specialized in the recognition of microbes and stress signals (63). In the brain, PRRs can promote pro-inflammatory cytokine production upon recognition of “neurodegeneration associated molecular patterns” (NAMPs) that consists in CNS-specific danger signals such as extracellular protein aggregates, molecules exposed by dying neurons, lipid degradation byproducts and myelin debris, among others (66). The most relevant PRRs associated to the development of PMDs are TLRs (Toll-like Receptors) and NLR (Nucleotide-binding domain, leucine-rich repeat containing) inflammasomes (63). These receptors are broadly expressed in CNS-myeloid cells including microglia, macrophages and infiltrating cells such as monocytes and dendritic cells (DCs) (63, 67). Interestingly, PRR-signaling and the UPR converge on several levels for amplification of inflammatory responses via activation of NF-kB, IRF-3, JNK and JAK/STAT modules (68–71). Signaling via TLR2 and TLR4 induces ER stress in peripheral macrophages and activates IRE1α and XBP1s, which in turn is required to increase production of IL-6 and TNF, thus connecting activation of the IRE1α-XBP1s branch of the UPR with TLR-dependent pro-inflammatory programs (68). In the CNS, misfolded α-synuclein and Fibrillar Aβ, characteristic in patients with PD and AD, can be sensed by TLR1/2 and TLR4, further promoting inflammation (63) (Figure 2). Moreover, injection of lipopolysaccharide (LPS), an agonist of TLR4, into the *substantia nigra* induces dopaminergic neuronal death resembling animal PD models (73). LPS-induced neurotoxicity and LPS-derived inducible nitric oxide synthase (iNOS) expression was shown to be mediated by the UPR related chaperone BiP/Grp78 and NF-kB (74, 75). Correspondingly, *Tlr4* null mice are protected from PD in a mouse model induced with neurotoxins (63, 76). Overall, TLR pathways activating the IRE1α-XBP1s axis are relevant drivers of PMDs, although the precise contribution of this UPR branch to TLR-induced neuroinflammation remains to be formally demonstrated.

Another PRR relevant in neurodegeneration modulated by the UPR, is the NLRP3 (NLR Family Pyrin Domain-Containing-3) inflammasome, a multimeric protein complex composed of the NLRP3 sensor, the adaptor ASC and activated caspase 1, which mediates the proteolytic activation of IL-1β and IL-18 and promotes a type of inflammatory cell death referred to as pyroptosis (63). In the brain, the NLRP3 inflammasome is activated by amyloid β and α-synuclein aggregates (63). The relevance of this protein complex is underscored by studies with *Nlrp3* deficient mice carrying mutations associated with familiar AD, which are protected from the disease (77). On a mechanistic level, the interplay between the UPR and inflammasome activation has been connected to IRE1α signaling (78), where the RNase domain of IRE1α increases the expression of TXNIP, an activator of the NLRP3 inflammasome, through degradation of the TXNIP-destabilizing microRNA miR-17 (78) (Figure 2). Considering the relevance of the NLRP3 inflammasome in AD progression and its dependence on IRE1α endonuclease, it is tempting to speculate that IRE1α activation in CNS-resident myeloid cells may contribute to the development of AD (79–84). Additionally, the B-class scavenger receptor CD36, upon recognition of amyloid β fibrils, forms a complex with TLR4/6, which triggers activation of the NLRP3 inflammasome, promoting cytokine and ROS production (67, 85).

On the other hand, in models of peripheral nerve damage, XBP1 expression has been shown to enhance nerve regeneration after injury, involving increased expression of the chemokine MCP-1 and macrophage infiltration, essential to remove myelin debris and allow axonal regeneration (44). PERK expression correlates with astroglial activation and production of IL-6 and the chemokines CCL2 and CCL20, which promotes microglial activation (71, 86). In spinal cord injury, ATF4 deficiency reduced microglial activation, which is associated with altered levels of IL-6, TNFα, and IL-1β (44–49). Similarly, ATF6 deficiency in the context of PD induced by neurotoxins leads to suppression of astroglial activation and decreased production of BDNF and anti-oxidative genes, such as heme oxygenase-1 (HO-1) and xCT (56). To sum up, ER stress and inflammation are both prevalent in many neurodegenerative diseases and NAMPs can alter neuronal function as well as promote inflammation through the activation of innate defense mechanisms of immune cells in the CNS, which can be modulated by UPR activity and vice versa.

### Immune targets of the UPR in the central nervous system

Although it is clear that inflammation contributes to neurodegeneration (61), there has been limited knowledge about the homeostasis of immune cells residing in the CNS. Recent technological advances in single cell analysis have provided insights into the identification and characterization of the vast diversity of immune cell lineages present in the healthy and pathogenic brain (61, 62). The potential role of the UPR in immune cell lineages in the CNS is illustrated in Figure 2.

#### Microglia

Microglia is the CNS-resident macrophage and most prominent myeloid cell in the brain (87). Microglia fine-tunes the development of neuronal circuits, neurogenesis and synaptic plasticity through the production of neurotrophic factors (88, 89). Given that several PRRs that signal via IRE1α and XBP1s such as TLR1/2 and TLR4, the NLRP3 inflammasome and nucleic acid sensors are expressed in this cell lineage, it is plausible that microglial XBP1s activation may contribute to the initiation of neuroinflammation. The ATF6 branch has also been associated with microglial activation and production of inflammatory mediators via NF-kB (90). Furthermore, although long conceived as a homogeneous cell type that becomes destructive in neurodegeneration (62), comprehensive single cell RNA analysis has demonstrated that a subset known as “disease-associated microglia” plays an important role in several CNS diseases including AD, ALS, MS and also in aging (62, 91–93). Thus, it is vital to elucidate whether protective microglial populations engage the UPR upon innate recognition of NAMPs, and whether microglial UPR is an intrinsic mechanism of sensing danger in the CNS.

#### Border associated macrophages

Border associated macrophages (BAMs) are a recently characterized population distinct from microglia and from infiltrating monocyte-derived macrophages, which display high heterogeneity and are classified per phenotype, development and location in the CNS (62, 94). Single cell analysis, fate mapping and parabiosis experiments revealed that these cells express distinct surface markers and differentially populate the pia mater, perivascular space, choroid plexus and dura mater (62, 94). Most of these subsets sample the environment, clear apoptotic cells and amyloid β plaques, and help maintaining CNS homeostasis in steady state. Up to date, there is no evidence available on the extent of UPR activation in BAMs. However, it has been described that splenic F4/80 macrophages display basal levels of IRE1α RNase activity and upon bacterial infection, peripheral macrophages induce XBP1s for enhancing cytokine production in a mechanism mediated by TLRs and reactive oxygen species (68, 95). However, whether CNS macrophages show a functional analogy to peripheral macrophages and also engage the IRE1α-XBP1s branch upon recognition of NAMPs (68) remains undetermined.

#### Dendritic cells

DCs are major APCs in the CNS, acting as sentinels between brain and periphery (87, 96–99). Steady-state CNS is populated by most DC subtypes, including plasmacytoid DCs (pDCs), and conventional DC type 1 (cDC1) and type 2 (cDC2) (62). These cells locate in the choroid plexus, pia mater and dura mater, but not in the perivascular space, suggesting that these compartments may serve as entry sites for MHC-dependent T cells (62, 96, 97). Importantly, DCs are key targets of the UPR. XBP1s is constitutively expressed by DCs and high XBP1s is a hallmark of cDC1s across tissues, although the CNS remains to be examined (95, 100, 101). Furthermore, cDC1s activate the IRE1α -XBP1 axis for development, survival in mucosal tissues and cross-presentation of antigens to CD8^+^ T cells, which may be of relevance in infections with neurotropic viruses (2, 102). In addition, cDC1s are highly sensitive to perturbations in XBP1 signaling and counter activate RIDD upon XBP1 loss (95, 101). The implication of RIDD and XBP1s signaling in DC subtypes in the CNS has not been explored so far but relevant aspects downstream of XBP1s and RIDD may encompass cytokine production upon recognition of protein aggregates, cell survival and cross-presentation of antigens to CD8^+^ T cells.

#### Lymphocytes

T and B cells survey the steady-state CNS exerting a neuroprotective role, but can become pathogenic under unresolved inflammation (57, 103–106). T cell numbers have been found to be increased in AD, PD, ALS and MS, and to contribute both to inflammation and neuronal dysfunction as well as to deferring inflammatory responses leading to nerodegeneration (107, 108). The immune response elicited by these cells in the CNS depends on their functional phenotype, although observations regarding cell number and T cell subset involved varies between different disease types and model of study (108–113). UPR activation in T cells is not completely elucidated, however the IRE1α-XBP1s branch has shown to regulate cell differentiation and cytokine production in CD8^+^ and CD4^+^ T cells under infection and chronic ER stress (114–118). During neuroinflammation and aging, B cells play a pathogenic role by producing pro-inflammatory cytokines, promoting effector T cells and activating macrophages via Fc receptors (62, 119–123). B cell development, activation and differentiation is critically regulated by IRE1α-XBP1s and ATF6, whilst absence of PERK favors plasma cell differentiation and immunoglobulin synthesis (124–128).

Overall, as proposed on Figure 2, activation of UPR components could occur in CNS-residing and infiltrating immune cells upon PRR recognition of protein aggregates, or due to noxious threats. The IRE1α-XBP1s axis has a key role in immune cell development from hematopoietic progenitors, cell survival and effector function, and it could be activated by NAMPs through PRR signaling in microglia, macrophages or dendritic cells, inducing cell maturation and activation (66, 68, 88, 97). The PERK pathway in contrast, is mostly deactivated to allow immune cells to fulfill their function under different inflammatory settings without going through apoptosis. In AD or PD however, sustained stimulation triggered by amyloid β or α-synuclein aggregates could lead to a dysfunctional activated phenotype associated to defective clearance and increased production of inflammatory mediators. This process could, in turn, attract more immune cells that exert a neurotoxic effect, promoting the accumulation of more protein aggregates, axonal destruction and neuronal malfunction (129, 130). Under this chronic ER stress, UPR signaling would be expected to be highly activated in CNS-related immune cells, in line with observations in brain samples of patients. Nevertheless, it remains to be addressed whether the UPR output in CNS-associated immune cells proves to be beneficial or detrimental for the development of PMDs, as is the case of neurons and astrocytes (131, 132).

## Concluding remarks

The interplay between the UPR, the immune system and the CNS in neurodegenerative diseases remains in its early stages. Intensive research will be required to accurately understand the role of ER stress in the immune-related aspects of CNS pathology and to determine whether UPR signaling in immune cells answers to a homeostatic or a terminal fate. It is also important to keep in mind the potential differences between human and mice immune cell types, since most knowledge gained in this matter emerges from studies in murine models. Through our knowledge on the UPR role in peripheral immunity and neurodegeneration models, better access to human samples and the advent of novel analytic tools for identification of the diversity of cell lineages, the cell-specific contribution of the UPR to neural and CNS-associated immune cells will begin to be elucidated, generating valuable knowledge that may provide therapeutic opportunities.

## Author contributions

All authors read and approved the final version of the manuscript. PG-G and FC-M contributed equally to the work. PG-G, FO, FC-M, and CH participated in manuscript conception and design.

### Conflict of interest statement

The authors declare that the research was conducted in the absence of any commercial or financial relationships that could be construed as a potential conflict of interest.
